# Maternal glycaemia and child educational attainment at age 5 in the Born in Bradford cohort

**DOI:** 10.1111/dme.70317

**Published:** 2026-04-05

**Authors:** Claire A. Wilson, Gillian Santorelli, Emily Simonoff, Louise M. Howard, Khalida Ismail, Rebecca M. Reynolds

**Affiliations:** ^1^ Department of Psychological Medicine, Institute of Psychiatry, Psychology and Neuroscience King‘s College London London UK; ^2^ South London and Maudsley NHS Foundation Trust London UK; ^3^ Born in Bradford, Bradford Teaching Hospitals NHS Foundation Trust Bradford UK; ^4^ Department of Child and Adolescent Psychiatry, Institute of Psychiatry, Psychology and Neuroscience King‘s College London London UK; ^5^ King‘s Women‘s Mental Health, Institute of Psychiatry, Psychology and Neuroscience King‘s College London London UK; ^6^ Centre for Cardiovascular Science University of Edinburgh Edinburgh UK

**Keywords:** diabetes in pregnancy, educational attainment, gestational diabetes

## Abstract

**Aims:**

There is emerging evidence that maternal hyperglycaemia may be associated with adverse offspring neuro‐behavioural outcomes, which are foundational to educational success. We hypothesised that higher levels of maternal glucose would be associated with poorer educational attainment in early childhood.

**Methods:**

The sample included 13,627 children from the UK's Born in Bradford cohort. Exposures included maternal fasting glucose, 2‐h post‐load glucose and a clinical diagnosis of gestational diabetes. The primary outcome was failure to achieve a ‘good level of development’ on the Early Years Foundation Stage Profile at age five. The association was tested using multivariable Poisson regression, accounting for sibling clusters and using multiple imputation for missing data.

**Results:**

Higher maternal fasting glucose (at 26–28 weeks gestation) was associated with an increased risk of failing to achieve a good level of development (adjusted RR 1.04; 95% CI 1.00, 1.08; *p* = 0.034). This association appeared stronger in children of Pakistani ethnicity compared to White British ethnicity. No association was found for 2‐h post‐load glucose or gestational diabetes diagnosis.

**Conclusions:**

These findings offer insights into the developmental origins of inequalities in child educational outcomes and highlight potential opportunities to optimise future health and learning through interventions during pregnancy.

**What's new?**

**What is already known?**

- Gestational diabetes is common and is associated with adverse child neuro‐behavioural outcomes.

**What has this study found?**

- The observed association between higher levels of maternal fasting glucose and poorer educational attainment in children at age five adds to existing evidence that there may be a dose–response relationship between maternal glycaemia and adverse outcomes.

**What are the implications of the study?**

- Our findings should inform the ongoing debate surrounding the criteria for screening, diagnosis and management of impaired glucose tolerance during pregnancy.

## 1 INTRODUCTION

Gestational diabetes (GDM) is defined as ‘glucose intolerance with onset during pregnancy’. Its global prevalence may be up to 30% depending on the diagnostic criteria employed and population studied.^1^ GDM is associated with adverse outcomes for the children, including an increased risk for a range of metabolic disorders, such as Type 2 diabetes and obesity.^1^ There has been substantially less focus on neuro‐behavioural offspring outcomes but emerging evidence suggests negative sequalae, which may in turn impact a child's readiness for school and later educational success.

Early studies of small samples of primary school‐age children exposed to GDM in utero have reported lower IQ scores and reduced gross and fine motor achievements than unexposed children.^2^, ^3^ However, these were most prominent in younger children (around 9 years of age) and differences between children born to mothers with or without GDM tended to diminish with age. There are fewer studies of older school‐aged children, but some find evidence for an association between GDM exposure and Autism Spectrum Disorders (ASD), Attention‐Deficit Hyperactivity Disorder (ADHD) and other neurodevelopmental disorders.^4^

There is a well‐documented linear relationship between maternal glucose and adverse pregnancy outcomes; this was demonstrated in the Hyperglycaemia and Adverse Outcome (HAPO) study of around 25,000 pregnant individuals across nine countries, for outcomes such as macrosomia and neonatal hypoglyaemia.^5^ Indeed other cohorts of school‐age children have observed an inverse correlation between maternal glycaemia and both IQ scores^6^ and the degree of fine and gross motor deficiency.^7^

Few studies have investigated the extent to which these early developmental delays may translate into educational outcomes. We hypothesised that increased levels of maternal glycaemia would be associated with poorer offspring educational attainment at age 5 years. We tested this hypothesis in Born in Bradford (BiB): a large prospective longitudinal cohort in the UK with a high prevalence of GDM.

## 2 METHODS

A directed acyclic graph (DAG) is provided in supplementary material (Figure S1).

### 2.1 Sample

BiB is a prospective longitudinal cohort of 12,450 individuals with 13,758 pregnancies in Bradford, UK. Bradford has high levels of deprivation; 60% of babies born in Bradford are born into the poorest 20% of the English population according to the Index of Multiple Deprivation (IMD 2010). Its ethnic makeup is predominantly bi‐ethnic: Pakistani and White British.^8^

Pregnant individuals were recruited to BiB during a routine appointment for a 2 h 75 g oral glucose tolerance test (OGTT), as currently recommended by the UK's National Institute for Health and Care Excellence (NICE) as the gold standard for diagnosing GDM. The test was offered to all participants between 26 and 28 weeks gestation. The sample used for analysis was all live births in the BiB cohort (Figure 1), excluding those with pre‐gestational (Type 1 and Type 2) diabetes.

**FIGURE 1:**
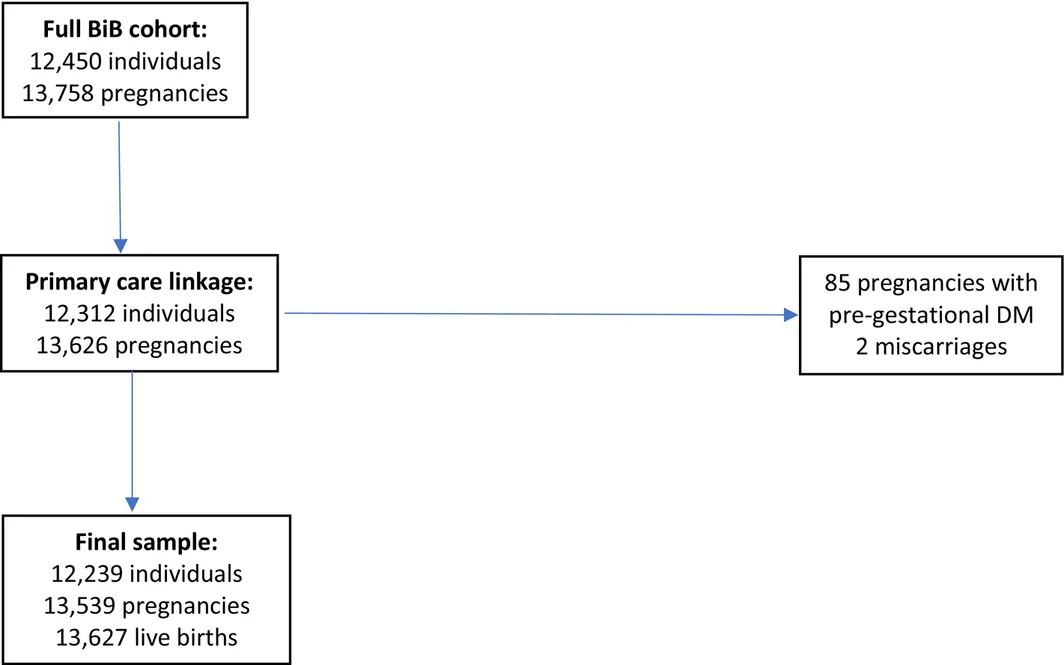
How the sample was obtained.

### 2.2 Exposure: Measures of maternal glycaemia

The primary exposure was the continuous variable of fasting glucose. Two‐hour post‐load glucose on the OGTT was used as a secondary variable. A diagnosis of GDM and its management was included as an additional secondary exposure. In BiB, GDM was diagnosed according to modified World Health Organisation (WHO) criteria (either fasting glucose ≥6.1 mmol/L or 2‐h post‐load glucose ≥7.8 mmol/L). A three‐category variable was used of: (1) no GDM, (2) GDM treated without insulin and (3) insulin‐treated GDM.

### 2.3 Outcome: Failure to meet a good level of development on the early years foundation stage profile

There is linkage within the BiB cohort to the child's education records, providing the results of national examinations. The earliest national collection of data on educational progress from school‐aged children in England is the Early Years Foundation Stage Profile (EYFSP), completed at the end of reception year (the first year of primary school) when children are aged 5 years. Teachers are asked to rate children across a number of different aspects of learning, based on their knowledge and accumulating observations of the child throughout the school year.

The most widely used indicator of child development at this age is a ‘good level of development’ (GLD) on the EYFSP, which is being rated by the teacher as ‘expected’ or ‘exceeding’ expectations across five of the areas covered by the EYFSP: (1) communication and language, (2) literacy, (3) mathematics, (4) physical development, (5) personal, social and emotional development.^9^ This binary variable of failure to attain GLD (yes or no) was used as the primary outcome.

### 2.4 Confounders

There were a number of variables identified as potential confounders due to their association with both GDM and child educational attainment but not on the causal pathway from exposure to outcome.^10^ These were maternal age at the time of the OGTT, tobacco smoking during pregnancy, singleton versus multiple pregnancy and other obstetric complications (as a binary variable of pre‐eclampsia and/or gestational hypertension). Maternal pre‐pregnancy body mass index (BMI) was also included, which was a continuous variable of BMI at pregnancy booking. Moreover, rather than using an index of deprivation to assign socioeconomic status, a five‐category variable of maternal education was used as a proxy because education is a more stable indicator of socioeconomic position than income, employment or area‐based deprivation and because the high levels of deprivation in BiB result in a highly skewed index of multiple deprivation distribution with limited discriminatory power.

There is also some evidence that the common mental disorders of depression and anxiety (CMD) may be associated with GDM^11^ and child neuro‐behavioural outcomes so CMD occurring prior to pregnancy (‘preconception’ CMD) was also included as a potential confounder. Consent was obtained for linkage to primary care (GP) records, which are held on SystmOne: a clinical system used by all practices in Bradford. These records provided information on medication prescriptions and diagnoses from that individual's birth to the date of conception of that individual's first ever pregnancy; these methods have been published elsewhere.^12^

### 2.5 Statistical analysis

Data were analysed using Stata version 18. Multivariable robust Poisson regression was employed to examine the association between the measures of maternal glycaemia and failure to attain GLD, within a generalised estimating equation framework using the robust sandwich estimator for standard errors to account for clustering from those individuals with more than one child in the sample (either due to twin or triplet pregnancies or multiple pregnancies occurring during the data collection period). Two analyses were performed: unadjusted and adjusted for the confounders described above to produce risk ratios (RRs) with 95% confidence intervals (CIs).

Multiple imputation by chained equations was implemented to handle missing data.^13^ 46 imputations were used to reflect the proportion of participants with any missing data across exposure, outcome and confounder variables.^14^ All analysis variables were included in the imputation model and an additional variable of being in receipt of child benefit payments or not was used as an auxiliary variable, as it was associated with being an incomplete case. Estimates were obtained by pooling results using Rubin's rules.^15^ Complete case analyses were also conducted.

### 2.6 Subgroup analyses

The above analyses were stratified:

1. Into two groups of Pakistani and White British maternal ethnicity. As these were the most prevalent ethnicities, further categories of ethnicity such as ‘Other’ would have been too heterogeneous.
2. By sex of the child

### 2.7 Sensitivity analyses

The above analyses were performed in each of the five EYFSP sub‐domains described above, in the full (unstratified) sample.

### 2.8 Ethics committee approval

Ethical approval for data collection in BiB was granted by Bradford Research Ethics Committee (Ref 07/H1302/112).

## 3 RESULTS

The demographics of BiB are broadly representative of Bradford as a whole.^8^ Table 1 displays the characteristics of the sample used for analysis and the proportion of missing data in each variable. Table S1 shows the characteristics of those with missing data (across exposure, outcome and confounder variables) versus no missing data. 1051 children were known to have been exposed to maternal GDM; 53% of the GDM cases were treated with insulin. 45% of the children with data on maternal ethnicity were Pakistani. Mean maternal age was 27 years. 75% of children with data on maternal education were from those with no higher education beyond secondary/high school (equal to or less than English A level). 46% of children with available data failed to meet a good level of development (GLD) on the EYFSP.

**TABLE 1:** Characteristics of the complete BiB sample (*N* = 13,627 children).

	*n*	%
Maternal fasting glucose (mmol/L)		
Mean (SD)	4.53 (0.6)	
Missing	1358	10.0
Maternal glucose 2 h post‐load (mmol/L)		
Mean (SD)	5.69 (1.5)	
Missing	1375	10.1
Maternal GDM		
No GDM	12,179	89.4
GDM treated without insulin	497	3.7
Insulin‐treated GDM	554	4.1
Missing	397	2.9
Maternal ethnicity		
Pakistani	5120	37.6
White British	4469	32.8
Other	1716	12.6
Missing	2322	17.0
Maternal age (years)		
Mean (SD)	27.32 (5.6)	
Missing	1355	9.9
Maternal education		
Less than 5 GCSE equivalents	2436	17.9
5 GCSE equivalents	3475	25.5
A level equivalents	1637	12.0
Higher than A level	2879	21.1
Other	872	6.4
Missing	2328	17.1
Multiple pregnancy		
Singleton	13,004	95.4
Multiple pregnancy (twins or triplets)	313	2.3
Missing	310	2.3
Maternal tobacco smoking in pregnancy		
Yes	1862	13.7
No	9445	69.3
Missing	2320	17.0
Obstetric complication (pre‐eclampsia and/or gestational hypertension)		
Yes	777	5.7
No	11,958	87.8
Missing	892	6.6
Maternal BMI		
Mean (SD)	26.03 (5.7)	
Missing	3130	23.0
Sex of child		
Male	5927	43.5
Female	5591	41.0
Missing	2109	15.5
Maternal indicator of preconception CMD		
Yes	2151	15.8
No	10,156	74.5
Missing	1320	9.7
Failure to meet good level of development (GLD) on EYFSP		
Yes	4801	35.2
No	5617	41.2
Missing	3209	23.6

There was evidence for an association between the primary exposure of fasting glucose and failure to attain GLD (unadjusted RR 1.04; 95% CI 1.00, 1.08; *p* = 0.026; adjusted RR 1.04; 95% CI 1.00, 1.08; *p* = 0.034) (Table 2). There was no evidence of an association between failure to attain GLD and 2‐h post‐load glucose on the OGTT on either unadjusted or adjusted analyses (adjusted RR 1.00; 95% CI 0.98, 1.01; *p* = 0.635). Likewise, there was no evidence of an association between a maternal diagnosis of GDM and failure to attain GLD (for GDM treated without insulin compared to no GDM adjusted RR 1.04; 95% CI 0.93, 1.16; *p* = 0.534 and for GDM treated with insulin compared to no GDM adjusted RR 0.98; 95% CI 0.88, 1.09; *p* = 0.679). However, the direction of effect differed between GDM treated with insulin compared to GDM not treated with insulin, with GDM treated with insulin showing a reduced risk of failure to attain GLD, albeit this was not significant. Results of complete case analyses were consistent with the imputed analyses, with similar effect estimates and overlapping confidence intervals (Table S2), indicating that the findings were robust to the handling of missing data.

**TABLE 2:** Associations between maternal glycaemia and EYFSP results (*N* = 13,627 children).

	Failure to attain good level of development (GLD) on EYFS**P**		
		RR (95% CI)	*p*
Unadjusted			
Maternal fasting glucose (mmol/L)			
Mean (SD)	4.54 (0.6)	1.04 (1.00, 1.08)	0.026
Maternal glucose 2 h post‐load (mmol/L)			
Mean (SD)	5.66 (1.5)	0.99 (0.98, 1.01)	0.393
Diagnosis of GDM			
Reference category = no GDM	45.8%		
GDM treated without insulin	47.5%	1.03 (0.92, 1.15)	0.602
Insulin‐treated GDM	44.3%	0.97 (0.87, 1.07)	0.543
Adjusted^a^			
Maternal fasting glucose (mmol/L)			
Mean (SD)	4.54 (0.6)	1.04 (1.00, 1.08)	0.034
Maternal glucose 2 h post‐load (mmol/L)			
Mean (SD)	5.66 (1.5)	1.00 (0.98, 1.01)	0.635
Diagnosis of GDM			
Reference category = no GDM	45.8%		
GDM treated without insulin	47.5%	1.04 (0.93, 1.16)	0.534
Insulin‐treated GDM	44.3%	0.98 (0.88, 1.09)	0.679

*Note*: Models using imputed data in a Poisson regression within a generalised estimating equation framework with robust standard errors.

^a^ Adjusted for maternal age, education, BMI, preconception common mental disorders, multiple pregnancy, tobacco smoking in pregnancy and obstetric complications.

### 3.1 Subgroup analyses

Table 3 displays the unadjusted and adjusted RRs for failure to attain GLD in children for each of the three measures of maternal glycaemia stratified by maternal ethnicity. Effect estimates differed between strata. There was stronger evidence for an association between increased levels of fasting glucose and increased risk of failure to attain GLD in the Pakistani ethnic subgroup than the White British, although this was attenuated to non‐significance on adjustment for confounders. In the primary analyses, a reduced (non‐significant) risk associated with insulin‐treated GDM (versus no GDM) was observed in the White British subgroup, which was not observed in the Pakistani subgroup.

**TABLE 3:** Associations between maternal glycaemia and EYFSP results stratified by maternal ethnicity.

	Pakistani (*N* = 5120 children)			White British (*N* = 4469 children)		
	Failure to attain good level of development (GLD) on EYFSP			Failure to attain good level of development (GLD) on EYFSP		
		RR (95% CI)	*p*		RR (95% CI)	*p*
Unadjusted						
Maternal fasting glucose (mmol/L)						
Mean (SD)	4.63 (0.7)	1.05 (1.00, 1.10)	0.042	4.40 (0.4)	0.97 (0.88, 1.08)	0.594
Maternal glucose 2 h post‐load (mmol/L)						
Mean (SD)	5.90 (1.7)	1.01 (0.99, 1.02)	0.575	5.37 (1.3)	0.95 (0.92, 0.99)	0.007
Diagnosis of GDM						
Reference category = no GDM	49.2%			39.8%		
GDM treated without insulin	47.5%	0.96 (0.82, 1.12)	0.586	46.2%	1.18 (0.86, 1.61)	0.307
Insulin‐treated GDM	49.2%	1.00 (0.88, 1.14)	0.993	37.8%	0.94 (0.73, 1.22)	0.651
Adjusted^a^						
Maternal fasting glucose (mmol/L)						
Mean (SD)	4.63 (0.7)	1.04 (0.99, 1.09)	0.127	4.40 (0.4)	0.99 (0.89, 1.10)	0.882
Maternal glucose 2 h post‐load (mmol/L)						
Mean (SD)	5.90 (1.7)	1.00 (0.98, 1.02)	0.717	5.37 (1.3)	0.96 (0.93, 1.00)	0.041
Diagnosis of GDM						
Reference category = no GDM	49.2%			39.8%		
GDM treated without insulin	47.5%	0.96 (0.82, 1.12)	0.573	46.2%	1.28 (0.94, 1.74)	0.112
Insulin‐treated GDM	49.2%	0.99 (0.87, 1.13)	0.875	37.8%	0.99 (0.76, 1.28)	0.926

*Note*: Models using imputed data in a Poisson regression within a generalised estimating equation framework with robust standard errors.

^a^ Adjusted for maternal age, education, BMI, preconception common mental disorders, multiple pregnancy, tobacco smoking in pregnancy and obstetric complications.

Table 4 displays the results stratified by sex of the child. For those with complete data on child sex and EYFSP results, 54.4% of males failed to achieve GLD versus 37.5% of females (*p* < 0.001 on chi square). While a formal test for interaction was not significant (*p* = 0.671), the adjusted association between maternal fasting glucose and failure to attain GLD may have been driven by females (adjusted RR 1.07; 95% CI 1.01, 1.14), compared to adjusted RR in males of 1.03 (95% CI 0.99, 1.08).

**TABLE 4:** Associations between maternal glycaemia and EYFSP results stratified by sex of child.

	Male (*N* = 5927 children)			Female (*N* = 5591 children)		
	Failure to attain good level of development (GLD) on EYFSP			Failure to attain good level of development (GLD) on EYFSP		
		RR (95% CI)	*p*		RR (95% CI)	*p*
Unadjusted						
Maternal fasting glucose (mmol/L)						
Mean (SD)	4.54 (0.6)	1.04 (1.00, 1.08)	0.071	4.55 (0.6)	1.05 (0.99, 1.12)	0.118
Maternal glucose 2 h post‐load (mmol/L)						
Mean (SD)	5.67 (1.5)	1.01 (0.99, 1.02)	0.499	5.67 (1.6)	0.99 (0.96, 1.01)	0.286
Diagnosis of GDM						
Reference category = no GDM	54.2%			37.7%		
GDM treated without insulin	58.3%	1.07 (0.95, 1.21)	0.279	37.4%	0.99 (0.82, 1.21)	0.950
Insulin‐treated GDM	55.6%	1.03 (0.92, 1.16)	0.626	34.3%	0.91 (0.76, 1.10)	0.336
Adjusted^a^						
Maternal fasting glucose (mmol/L)						
Mean (SD)	4.54 (0.6)	1.03 (0.99, 1.08)	0.162	4.55 (0.6)	1.07 (1.01, 1.14)	0.034
Maternal glucose 2 h post‐load (mmol/L)						
Mean (SD)	5.67 (1.5)	1.01 (0.99, 1.02)	0.387	5.67 (1.6)	0.99 (0.97, 1.02)	0.659
Diagnosis of GDM						
Reference category = no GDM	54.2%			37.7%		
GDM treated without insulin	58.3%	1.07 (0.94, 1.21)	0.295	37.4%	1.02 (0.84, 1.25)	0.817
Insulin‐treated GDM	55.6%	1.03 (0.92, 1.16)	0.626	34.3%	0.97 (0.80, 1.17)	0.734

*Note*: Models using imputed data in a Poisson regression within a generalised estimating equation framework with robust standard errors.

^a^ Adjusted for maternal age, education, BMI, preconception common mental disorders, multiple pregnancy, tobacco smoking in pregnancy and obstetric complications.

### 3.2 Sensitivity analyses

The results of the analyses for each of the five domains of the EYFSP are provided in Tables S3–S7 (supplementary material). For those with EYFSP results, 25% of children failed to meet GLD in communication and language, 37.4% in literacy, 32.9% in mathematics, 18.6% in physical development and 22.4% in personal, social and emotional development. While the results of these analyses of the sub‐domains of the EYFSP were similar to that of the global measure used for the primary analyses, there was somewhat more evidence of an association between fasting glucose and communication and language (unadjusted RR 1.06; 95% CI 1.00, 1.12; *p* = 0.038; adjusted RR 1.06; 95% CI 0.99, 1.12; *p* = 0.078) and physical development (unadjusted RR 1.08; 95% CI 1.00, 1.16; *p* = 0.036; adjusted RR 1.06; 95% CI 0.98, 1.14; *p* = 0.160) than literacy (unadjusted RR 1.04; 95% CI 1.00, 1.09; *p* = 0.062; adjusted RR 1.03; 95% CI 0.99, 1.08; *p* = 0.146), mathematics (unadjusted RR 1.04; 95% CI 0.99, 1.09; *p* = 0.119; adjusted RR 1.04; 95% CI 0.99, 1.09; *p* = 0.135) or personal, social and emotional development (unadjusted RR 1.05; 95% CI 0.99, 1.12; *p* = 0.117; adjusted RR 1.05; 95% CI 0.98, 1.12; *p* = 0.179).

## 4 DISCUSSION

### 4.1 Summary of findings in context

In this study, higher maternal fasting glucose was associated with an increased risk of offspring failure to achieve a good level of development (GLD) at age five (adjusted RR 1.04; 95% CI 1.00, 1.08; *p* = 0.034). To our knowledge, this is the first study to demonstrate such a linear relationship between maternal glucose and child educational attainment at age five. Reduced educational attainment at age 16 has been observed in another UK birth cohort (the Avon Longitudinal Study of Parents and Children (ALSPAC) cohort) for those children exposed to pre‐gestational diabetes (but not to GDM or maternal glycosuria); maternal glycosuria was associated with lower IQ at age eight.^16^ The ethnic composition of the ALSPAC cohort, which is predominantly White British, differs from that of BiB, which is bi‐ethnic (White British and Pakistani). In our subgroup analysis, the association between fasting glucose and poorer educational outcomes appeared stronger in children of Pakistani than White British ethnicity. We have shown, in previous analyses of BiB, that the educational attainment of children of Pakistani ethnicity may be more vulnerable to adversities such as GDM exposure in utero.^17^ However, subgroup analyses stratified by maternal ethnicity and child sex showed less evidence of associations than the primary analysis and the wider confidence intervals in the stratified models suggest reduced precision due to smaller sample sizes. It is also plausible that adjustment for socioeconomic factors, strongly patterned by ethnicity in BiB, contributed to the attenuation. Differences between child sex would be expected given the differential developmental programming often observed between sexes,^18^ including findings from a recent systematic review that offspring of female sex were more at risk of neurodevelopmental disorders following exposure to maternal Type 2 diabetes.^19^

Furthermore, there was no evidence of an association between the secondary exposures of 2 h post‐load glucose and GDM diagnosis and failure to attain GLD. However, it is important to note that the fasting diagnostic threshold for GDM in the BiB cohort was relatively high and the 2 h threshold relatively low compared to other diagnostic criteria for GDM, using the modified WHO criteria at the time.^20^ The (non‐significant) differing directions of effect seen for insulin treated (reduced risk of failure to attain GLD) versus non‐insulin treated GDM (increased risk) may reflect the positive effect of treatment or may merely reflect a population more engaged with and able to access effective GDM treatment; the somewhat greater reduction in risk with insulin treated GDM in the White British subgroup may support this hypothesis.

### 4.2 Potential mechanisms

Hyperglycaemia is associated with increased levels of oxidative stress, which is known to affect the developing brain^21^; epigenetic changes^22^ and neuroinflammation are putative underlying mechanisms.^23^ One of the brain areas most impacted appears to be the hippocampus, involved in learning and memory, explaining the increased risk for poorer educational attainment with higher levels of maternal glucose, although it is less clear what may explain the differences, albeit small, between EYFSP subdomains. In BiB, poorer EYFSP attainment is associated with ASD; ASD is also associated with exposure to GDM^24^ so this may explain the slightly stronger evidence of an association between fasting glucose and communication and language, which is a core area of impairment in ASD. Hyperglycaemia during pregnancy may also be associated with adverse outcomes in the postnatal period, such as perinatal mental disorders,^25^ which may serve to mediate the relationship with subsequent adverse neuro‐behavioural outcomes. The reasons why basal glucose state may be more detrimental to fetal neurodevelopment than post‐prandial spikes are unknown and require exploration in other datasets.

### 4.3 Strengths and limitations

Key strengths of this study include its large, bi‐ethnic cohort, which provides a novel opportunity to explore ethnic differences and the use of the EYFSP: a widely‐used and validated national outcome measure.^26^

However, the study also has important limitations. The cohort has high rates of consanguinity and congenital anomalies,^8^ which could independently impact educational attainment. Data on consanguinity are classified as highly sensitive information so were not available for use in this analysis. The findings may also have limited generalisability beyond this specific population, which is characterised by a predominantly bi‐ethnic (Pakistani and White British) composition and high levels of socioeconomic deprivation.

Moreover, loss of outcome data is minimised by using routinely collected data, which minimises some forms of attrition bias, although selection bias remains possible. School absence rates in state‐funded primary schools in Bradford (4.4%) are slightly higher than the national average (4%), suggesting that outcomes for the most vulnerable children may have been disproportionately missed.^27^

### 4.4 Implications and conclusions

The observed association between higher levels of maternal fasting glucose and poorer educational attainment in offspring has implications for future research, clinical practice and health policy. Our findings related to educational attainment add to the now well‐documented linear relationship between maternal glucose and adverse pregnancy outcomes.^5^, ^28^ There has been considerable debate about the diagnostic criteria for GDM and some have even argued that GDM may be more usefully conceptualised as a spectrum of maternal hyperglycaemia than a distinct disease.^29^ Intervention studies that focus on optimising maternal glucose should include educational outcome measures, in order to ascertain the extent to which this is associated with improvements in child outcomes. Further examination of the underlying mechanisms, including potential mediators for the relationship between maternal glycaemia and child educational outcomes, could illuminate future targets for intervention. Information about GDM treatment other than insulin was limited in BiB; for example, only 50 individuals are recorded as using metformin. This is an important consideration for future research studies given the evidence that metformin has a neuroprotective effect in the developing infant.^30^ Our findings also highlight potential ethnic inequalities in the early origins of child educational attainment, underpinning the importance of disaggregating data when examining developmental pathways^31^ in order to fully understand and optimise child development within diverse populations.

## FUNDING INFORMATION

Born in Bradford receives core infrastructure funding from the Wellcome Trust (WT101597M) and a joint grant from the UK Medical Research Council (MRC) and Economic and Social Science Research Council (ESRC) (MR/N024397/1). CAW carried out this work as part of a UK Medical Research Council (MRC) funded Clinical Research Training Fellowship (MR/P019293/1). RMR acknowledges the support of the British Heart Foundation (RE/18/5/34216). KI receives salary support from the National Institute for Health and Care Research (NIHR) Biomedical Research Centre at South London and Maudsley NHS Foundation Trust and King's College London.

## CONFLICT OF INTEREST STATEMENT

Authors declare no competing interests.

## Supporting information


**Table S1:** Characteristics of participants with missing data.
**Table S2:** Complete case analysis of associations between maternal glycaemia and EYFSP results (*N* = 7379 children).
**Table S3:** Associations between maternal glycaemia and EYFSP communication and language results (*N* = 13,627 children).
**Table S4:** Associations between maternal glycaemia and EYFSP literacy results (*N* = 13,627 children).
**Table S5:** Associations between maternal glycaemia and EYFSP mathematics results (*N* = 13,627 children).
**Table S6:** Associations between maternal glycaemia and EYFSP physical development results (*N* = 13,627 children).
**Table S7:** Associations between maternal glycaemia and personal, social and emotional development on EYFSP (*N* = 13,627 children).
**Figure S1:** Directed acyclic graph (DAG) for the relationship between maternal hyperglycaemia and child educational attainment.
