# Supplementary material for: Maternal glycaemia and child educational attainment at age 5 in the Born in Bradford cohort
Source: Diabet Med. 2026 Apr 5;43(10):e70317. doi: 10.1111/dme.70317 (PMC13643614; doi:10.1111/dme.70317)
Supplement: Supplementary file 1 [file DME-43-e70317-s001.docx]

**Maternal glycaemia and child educational attainment at age 5 in the Born in Bradford cohort**

**Supplementary material**

| **Table S1: Characteristics of participants with missing data** | | | |
| --- | --- | --- | --- |
|  | **Participants with no missing data N=7379** | **Participants with some missing data N=6248** | **p value*** |
|  | **n** | **n** |  |
| **Maternal fasting glucose (mmol/l)** |  |  |  |
| Mean (SD) | 4.52 (0.5) | 4.54 (0.6) | 0.022 |
| **Maternal glucose 2 hour post-load (mmol/l)** |  |  |  |
| Mean (SD) | 5.70 (1.5) | 5.68 (1.5) | 0.490 |
| **Maternal GDM** |  |  |  |
| No GDM | 6776 | 5403 | <0.001 |
| GDM treated without insulin | 221 | 276 |  |
| Insulin-treated GDM | 382 | 172 |  |
| **Maternal ethnicity** |  |  |  |
| Pakistani | 3537 | 1590 | <0.001 |
| White British | 2879 | 1583 |  |
| Other | 956 | 760 |  |
| **Maternal age (years)** |  |  |  |
| Mean (SD) | 27.34 (5.6) | 27.29 (5.6) | 0.609 |
| **Maternal education** |  |  |  |
| Less than 5 GCSE equivalents | 1643 | 793 | <0.001 |
| 5 GCSE equivalents | 2385 | 1090 |  |
| A level equivalents | 1100 | 537 |  |
| Higher than A level | 1714 | 1165 |  |
| Other | 537 | 335 |  |
| **Multiple pregnancy** |  |  |  |
| Singleton | 7190 | 5814 | 0.073 |
| Multiple pregnancy (twins or triplets) | 189 | 124 |  |
| **Maternal tobacco smoking in pregnancy** |  |  |  |
| Yes | 6151 | 3294 | 0.49 |
| No | 1228 | 634 |  |
| **Obstetric complication (pre-eclampsia and/or gestational hypertension)** |  |  |  |
| Yes | 456 | 321 | 0.664 |
| No | 6923 | 5035 |  |
| **Maternal BMI** |  |  |  |
| Mean (SD) | 26.04 (5.7) | 26.00 (5.7) | 0.753 |
| **Sex of child** |  |  |  |
| Male | 3724 | 2203 | 0.016 |
| Female | 3633 | 1958 |  |
| **Maternal indicator of preconception CMD** |  |  |  |
| Yes | 1332 | 852 | 0.278 |
| No | 6047 | 4076 |  |
| **Failure to meet good level of development on EYFSP** |  |  |  |
| Yes | 3320 | 1481 | <0.001 |
| No | 4059 | 1558 |  |
| **on chi square for categorical variables and t test for continuous variables* |  |  |  |

| **Table S2: Complete case analysis of associations between maternal glycaemia and EYFSP results (N=7379 children)** | | | | | |
| --- | --- | --- | --- | --- | --- |
|  | **Failure to attain good level of development (GLD) on EYFSP** | | | | |
|  |  |  |  |  |  |
| **Unadjusted** |  | **RR** | **(95% CI)** | | **p** |
| *Maternal fasting glucose (mmol/l)* |  |  |  |  |  |
| Mean (SD) | 4.54 (0.6) | 1.07 | (1.02, | 1.11) | 0.002 |
|  | | | | | |
| *Maternal glucose 2 hour post-load (mmol/l)* |  |  |  |  |  |
| Mean (SD) | 5.70 (1.6) | 1.00 | (0.98, | 1.02) | 0.999 |
|  | | | | | |
| *Diagnosis of GDM* Reference category= no GDM | 45.0% |  |  |  |  |
| GDM treated without insulin | 46.2% | 1.02 | (0.88, | 1.18) | 0.762 |
| Insulin-treated GDM | 44.2% | 0.99 | (0.88, | 1.11) | 0.863 |
|  | | | | | |
| **Adjusted*** |  | **RR** | **(95% CI)** | | **p** |
| *Maternal fasting glucose (mmol/l)* |  |  |  |  |  |
| Mean (SD) | 4.54 (0.6) | 1.07 | (1.02, | 1.11) | 0.002 |
|  | | | | | |
| *Maternal glucose 2 hour post-load (mmol/l)* |  |  |  |  |  |
| Mean (SD) | 5.70 (1.6) | 1.00 | (0.99, | 1.02) | 0.680 |
|  | | | | | |
| *Diagnosis of GDM* Reference category= no GDM | 45.0% |  |  |  |  |
| GDM treated without insulin | 46.2% | 1.05 | (0.91, | 1.22) | 0.473 |
| Insulin-treated GDM | 44.2% | 1.01 | (0.90, | 1.13) | 0.895 |
|  | | | | | |
| *Models using a Poisson regression within a generalised estimating equation framework with robust standard errors *adjusted for maternal age, education, BMI, preconception common mental disorders, multiple pregnancy, tobacco smoking in pregnancy and obstetric complications.* | | | | | |

| **Table S3: Associations between maternal glycaemia and EYFSP communication and language results (N=13,627 children)** | | | | | |
| --- | --- | --- | --- | --- | --- |
|  | **Failure to attain good level of development (GLD)** | | | | |
|  |  |  |  |  |  |
| **Unadjusted** |  | **RR** | **(95% CI)** | | **p** |
| *Maternal fasting glucose (mmol/l)* |  |  |  |  |  |
| Mean (SD) | 4.54 (0.6) | 1.06 | (1.00, | 1.12) | 0.038 |
|  | | | | | |
| *Maternal glucose 2 hour post-load (mmol/l)* |  |  |  |  |  |
| Mean (SD) | 5.67 (1.5) | 1.00 | (0.98, | 1.02) | 0.914 |
|  | | | | | |
| *Diagnosis of GDM* Reference category= no GDM | 24.8% |  |  |  |  |
| GDM treated without insulin | 26.1% | 1.04 | (0.87, | 1.25) | 0.643 |
| Insulin-treated GDM | 23.0% | 0.92 | (0.78, | 1.10) | 0.360 |
|  | | | | | |
| **Adjusted*** |  | **RR** | **(95% CI)** | | **p** |
| *Maternal fasting glucose (mmol/l)* |  |  |  |  |  |
| Mean (SD) | 4.54 (0.6) | 1.06 | (0.99, | 1.12) | 0.078 |
|  | | | | | |
| *Maternal glucose 2 hour post-load (mmol/l)* |  |  |  |  |  |
| Mean (SD) | 5.67 (1.5) | 1.00 | (0.97, | 1.02) | 0.850 |
|  | | | | | |
| *Diagnosis of GDM* Reference category= no GDM | 24.8% |  |  |  |  |
| GDM treated without insulin | 26.1% | 1.03 | (0.86, | 1.23) | 0.773 |
| Insulin-treated GDM | 23.0% | 0.93 | (0.78, | 1.10) | 0.392 |
|  | | | | | |
| *Models using imputed data in a Poisson regression within a generalised estimating equation framework with robust standard errors *adjusted for maternal age, education, BMI, preconception common mental disorders, multiple pregnancy, tobacco smoking in pregnancy and obstetric complications.* | | | | | |

| **Table S4: Associations between maternal glycaemia and EYFSP literacy results (N=13,627 children)** | | | | | |
| --- | --- | --- | --- | --- | --- |
|  | **Failure to attain good level of development (GLD)** | | | | |
|  |  |  |  |  |  |
| **Unadjusted** |  | **RR** | **(95% CI)** | | **p** |
| *Maternal fasting glucose (mmol/l)* |  |  |  |  |  |
| Mean (SD) | 4.54 (0.6) | 1.04 | (1.00, | 1.09) | 0.062 |
|  | | | | | |
| *Maternal glucose 2 hour post-load (mmol/l)* |  |  |  |  |  |
| Mean (SD) | 5.65 (1.5) | 0.99 | (0.97, | 1.01) | 0.282 |
|  | | | | | |
| *Diagnosis of GDM* Reference category= no GDM | 37.1% |  |  |  |  |
| GDM treated without insulin | 39.5% | 1.06 | (0.93, | 1.20) | 0.420 |
| Insulin-treated GDM | 33.3% | 0.90 | (0.78, | 1.02) | 0.100 |
|  | | | | | |
| **Adjusted*** |  | **RR** | **(95% CI)** | | **p** |
| *Maternal fasting glucose (mmol/l)* |  |  |  |  |  |
| Mean (SD) | 4.54 (0.6) | 1.03 | (0.99, | 1.08) | 0.146 |
|  | | | | | |
| *Maternal glucose 2 hour post-load (mmol/l)* |  |  |  |  |  |
| Mean (SD) | 5.65 (1.5) | 0.99 | (0.97, | 1.01) | 0.330 |
|  | | | | | |
| *Diagnosis of GDM* Reference category= no GDM | 37.1% |  |  |  |  |
| GDM treated without insulin | 39.5% | 1.06 | (0.93, | 1.20) | 0.419 |
| Insulin-treated GDM | 33.3% | 0.89 | (0.78, | 1.02) | 0.096 |
|  | | | | | |
| *Models using imputed data in a Poisson regression within a generalised estimating equation framework with robust standard errors *adjusted for maternal age, education, BMI, preconception common mental disorders, multiple pregnancy, tobacco smoking in pregnancy and obstetric complications.* | | | | | |

| **Table S5: Associations between maternal glycaemia and EYFSP mathematics results (N=13,627 children)** | | | | | |
| --- | --- | --- | --- | --- | --- |
|  | **Failure to attain good level of development (GLD)** | | | | |
|  |  |  |  |  |  |
| **Unadjusted** |  | **RR** | **(95% CI)** | | **p** |
| *Maternal fasting glucose (mmol/l)* |  |  |  |  |  |
| Mean (SD) | 4.54 (0.6) | 1.04 | (0.99, | 1.09) | 0.119 |
|  | | | | | |
| *Maternal glucose 2 hour post-load (mmol/l)* |  |  |  |  |  |
| Mean (SD) | 5.65 (1.5) | 0.99 | (0.97, | 1.01) | 0.229 |
|  | | | | | |
| *Diagnosis of GDM* Reference category= no GDM | 32.7% |  |  |  |  |
| GDM treated without insulin | 35.5% | 1.08 | (0.93, | 1.24) | 0.319 |
| Insulin-treated GDM | 29.0% | 0.88 | (0.76, | 1.02) | 0.096 |
|  | | | | | |
| **Adjusted*** |  | **RR** | **(95% CI)** | | **p** |
| *Maternal fasting glucose (mmol/l)* |  |  |  |  |  |
| Mean (SD) | 4.54 (0.6) | 1.04 | (0.99, | 1.09) | 0.135 |
|  | | | | | |
| *Maternal glucose 2 hour post-load (mmol/l)* |  |  |  |  |  |
| Mean (SD) | 5.65 (1.5) | 0.99 | (0.97, | 1.01) | 0.381 |
|  | | | | | |
| *Diagnosis of GDM* Reference category= no GDM | 32.7% |  |  |  |  |
| GDM treated without insulin | 35.5% | 1.08 | (0.94, | 1.25) | 0.273 |
| Insulin-treated GDM | 29.0% | 0.90 | (0.77, | 1.04) | 0.144 |
|  | | | | | |
| *Models using imputed data in a Poisson regression within a generalised estimating equation framework with robust standard errors *adjusted for maternal age, education, BMI, preconception common mental disorders, multiple pregnancy, tobacco smoking in pregnancy and obstetric complications.* | | | | | |

| **Table S6: Associations between maternal glycaemia and EYFSP physical development results (N=13,627 children)** | | | | | |
| --- | --- | --- | --- | --- | --- |
|  | **Failure to attain good level of development (GLD)** | | | | |
|  |  |  |  |  |  |
| **Unadjusted** |  | **RR** | **(95% CI)** | | **p** |
| *Maternal fasting glucose (mmol/l)* |  |  |  |  |  |
| Mean (SD) | 4.55 (0.6) | 1.08 | (1.00, | 1.16) | 0.036 |
|  | | | | | |
| *Maternal glucose 2 hour post-load (mmol/l)* |  |  |  |  |  |
| Mean (SD) | 5.68 (1.6) | 1.00 | (0.97, | 1.03) | 0.799 |
|  | | | | | |
| *Diagnosis of GDM* Reference category= no GDM | 18.3% |  |  |  |  |
| GDM treated without insulin | 20.6% | 1.10 | (0.90, | 1.37) | 0.326 |
| Insulin-treated GDM | 18.6% | 1.00 | (0.83, | 1.22) | 0.968 |
|  | | | | | |
| **Adjusted*** |  | **RR** | **(95% CI)** | | **p** |
| *Maternal fasting glucose (mmol/l)* |  |  |  |  |  |
| Mean (SD) | 4.55 (0.6) | 1.06 | (0.98, | 1.14) | 0.160 |
|  | | | | | |
| *Maternal glucose 2 hour post-load (mmol/l)* |  |  |  |  |  |
| Mean (SD) | 5.68 (1.6) | 1.00 | (0.97, | 1.03) | 0.922 |
|  | | | | | |
| *Diagnosis of GDM* Reference category= no GDM | 18.3% |  |  |  |  |
| GDM treated without insulin | 20.6% | 1.09 | (0.88, | 1.34) | 0.435 |
| Insulin-treated GDM | 18.6% | 0.99 | (0.81, | 1.20) | 0.907 |
|  | | | | | |
| *Models using imputed data in a Poisson regression within a generalised estimating equation framework with robust standard errors *adjusted for maternal age, education, BMI, preconception common mental disorders, multiple pregnancy, tobacco smoking in pregnancy and obstetric complications.* | | | | | |

| **Table S7: Associations between maternal glycaemia and personal, social and emotional development on EYFSP (N=13,627 children)** | | | | | |
| --- | --- | --- | --- | --- | --- |
|  | **Failure to attain good level of development (GLD)** | | | | |
|  |  |  |  |  |  |
| **Unadjusted** |  | **RR** | **(95% CI)** | | **p** |
| *Maternal fasting glucose (mmol/l)* |  |  |  |  |  |
| Mean (SD) | 4.54 (0.6) | 1.05 | (0.99, | 1.12) | 0.117 |
|  | | | | | |
| *Maternal glucose 2 hour post-load (mmol/l)* |  |  |  |  |  |
| Mean (SD) | 5.66 (1.5) | 1.00 | (0.97, | 1.02) | 0.741 |
|  | | | | | |
| *Diagnosis of GDM* Reference category= no GDM | 22.3% |  |  |  |  |
| GDM treated without insulin | 21.8% | 0.97 | (0.79, | 1.19) | 0.779 |
| Insulin-treated GDM | 20.5% | 0.92 | (0.76, | 1.10) | 0.348 |
|  | | | | | |
| **Adjusted*** |  | **RR** | **(95% CI)** | | **p** |
| *Maternal fasting glucose (mmol/l)* |  |  |  |  |  |
| Mean (SD) | 4.54 (0.6) | 1.05 | (0.98, | 1.12) | 0.179 |
|  | | | | | |
| *Maternal glucose 2 hour post-load (mmol/l)* |  |  |  |  |  |
| Mean (SD) | 5.66 (1.5) | 1.00 | (0.97, | 1.02) | 0.786 |
|  | | | | | |
| *Diagnosis of GDM* Reference category= no GDM | 22.3% |  |  |  |  |
| GDM treated without insulin | 21.8% | 0.97 | (0.79, | 1.19) | 0.755 |
| Insulin-treated GDM | 20.5% | 0.92 | (0.76, | 1.11) | 0.384 |
|  | | | | | |
| *Models using imputed data in a Poisson regression within a generalised estimating equation framework with robust standard errors *adjusted for maternal age, education, BMI, preconception common mental disorders, multiple pregnancy, tobacco smoking in pregnancy and obstetric complications.* | | | | | |

**Figure S1: Directed acyclic graph (DAG) for the relationship between maternal hyperglycaemia and child educational attainment**

Maternal age

Tobacco smoking during pregnancy

Multiple pregnancy (twins and triplets)

Other obstetric complications (pre-eclampsia and/or gestational hypertension)

Maternal BMI

Maternal education

Child educational attainment

Maternal hyperglycaemia
